# A complex multimodal activity intervention to reduce the risk of dementia in mild cognitive impairment–ThinkingFit: pilot and feasibility study for a randomized controlled trial

**DOI:** 10.1186/1471-244X-14-129

**Published:** 2014-05-05

**Authors:** Thomas M Dannhauser, Martin Cleverley, Tim J Whitfield, Ben (C) Fletcher, Tim Stevens, Zuzana Walker

**Affiliations:** 1 University College London, London, UK; 2 North Essex Partnership University NHS Foundation Trust, Chelmsford, Essex, UK; 3 University of Hertfordshire, Hatfield, Hertfordshire, UK; 4 Istanbul Bilgi University, Istanbul, Turkey

**Keywords:** Mild cognitive impairment, Alzheimer’s dementia, Dementia prevention, Complex activity intervention, Physical activity, Cognitive stimulation, Social stimulation

## Abstract

**Background:**

Dementia affects 35 million people worldwide and is currently incurable. Many cases may be preventable because regular participation in physical, mental and social leisure activities during middle age is associated with up to 47% dementia risk reduction. However, the majority of middle-aged adults are not active enough. MCI is therefore a clear target for activity interventions aimed at reducing dementia risk. An active lifestyle during middle age reduces dementia risk but it remains to be determined if increased activity reduces dementia risk when MCI is already evident. Before this can be investigated conclusively, complex multimodal activity programmes are required that (1) combine multiple health promoting activities, (2) engage people with MCI, and (3) result in sufficient adherence rates.

**Methods:**

We designed the ThinkingFit programme to engage people with MCI in a complex intervention comprised of three activity components: physical activity, group-based cognitive stimulation (GCST) and individual cognitive stimulation (ICST). Engagement and adherence was promoted by applying specific psychological techniques to enhance behavioural flexibility in an early pre-phase and during the course of the intervention. To pilot the intervention, participants served as their own controls during a 6- to 12-week run-in period, which was followed by 12 weeks of activity intervention.

**Results:**

Out of 212 MCI patients screened, 163 were eligible, 70 consented and 67 completed the intervention (mean age 74 years). Activity adherence rates were high: physical activity = 71%; GCST = 83%; ICST = 67%. Significant treatment effects (*p* < .05) were evident on physical health outcomes (decreased BMI and systolic blood pressure, [pre/post values of 26.3/25.9 kg/m^2^ and 145/136 mmHg respectively]), fitness (decreased resting and recovery heart rate [68/65 bpm and 75/69 bpm]), and cognition (improved working memory [5.3/6.3 items]).

**Conclusions:**

We found satisfactory recruitment, retention and engagement rates, coupled with significant treatment effects in elderly MCI patients. It appears feasible to conduct randomized controlled trials of the dementia prevention potential of complex multimodal activity programmes like ThinkingFit.

**Trial registration:**

ClinicalTrials.gov registration nr: NCT01603862; date: 17/5/2012.

## Background

At present there is no cure for the 35.6 million people living with dementia. However, regular participation in specific physical, cognitive and socially stimulating leisure activities during mid-life reduces the risk of dementia in later life by 28-47% [1-3]. This activity-associated risk reduction is probably due to the positive effects that specific activities have on known modifiable dementia risk factors that cause an estimated 50% of dementia and include physical- and cognitive-inactivity, obesity, hypertension and diabetes [4]. Currently 60% of people are not active enough to benefit and engaging people in regular activities is therefore a promising dementia prevention strategy [5]. This will require complex multimodal activity programmes that combine the most beneficial dementia prevention activities, are acceptable to people and result in long-term activity participation and therefore lifestyle change. The design of such programmes has not been determined yet and is a prerequisite to studying such interventions.

Preventative activity interventions can be targeted at high-risk groups such as patients diagnosed with Mild Cognitive Impairment (MCI). MCI is diagnosed in patients who (1) complain of cognitive deficits, (2) have impairment in one or more cognitive domain on testing after adjusting for age and educational attainment, (3) have preserved general cognitive function, (4) function at their usual level in their daily activities, (5) and do not meet diagnostic criteria for dementia [6]. MCI is often prodromal for the most prevalent dementia aetiologies, including Alzheimer’s disease, cerebrovascular disease and Lewy body disease. Meta-analysis reveals that 39% of patients diagnosed with MCI in specialist settings convert to dementia when followed up for a minimum of three years, compared to less than 1% in healthy elderly [7]. Strong associations exist between increased physical and mental activity in middle age and reduced risk of dementia in later life, and in MCI physical activity and [8-10] cognitive interventions [11] have resulted in improved cognition. However, it remains to be determined if taking part in complex multimodal activity programmes, that combines physical, social and mental stimulation, at the stage of MCI would reduce the risk of dementia. Evidence on the most effective methods to engage MCI patients in such complex activity programmes is scarce, whilst studies are underway to determine the efficacy of goal setting and mentoring to increase physical and cognitive activity in healthy elderly people and those with cardiac disease [12,13].

Physical, cognitive and social leisure activities appear to have the most beneficial effects on cognition and the most cognitively enhancing and protective activities have specific characteristics. The most beneficial physical activity programmes in healthy older adults included: combined aerobic fitness and strength training, duration of activities beyond 6 months, duration of training sessions between 30 and 45 minutes, and targeting females and people in the age range of 65 to 80 years [14,15]. The mechanisms responsible for the reduced risk associated with activity participation have not been clarified yet. Available evidence most strongly supports the beneficial effects of physical activity on cognition and these may be medicated via effects on physical resources, mental resources and chronic diseases or states [16,17]. Cognitively stimulating activities are also associated with reduced risk of cognitive decline in later life and more pronounced effects are related to increased complexity of activities and associated environments [18-20]. Social activities are associated with reduced dementia risk and socialising robustly stimulates memory, attention and executive processing, and it can be achieved by presenting cognitive and physical activities in small groups [21-24].

Current public health strategies of activity promotion have not succeeded in spite of the known benefits. The majority of over 65s do not participate in regular physical activity and have no intention to do so [5,25,26]. Important barriers to starting new activities have been identified and strategies to overcome these barriers have been developed and can lead to sustained increased levels of activity for up to two years [27]. Low activity participation rates have been ascribed to known barriers that include: (1) lack of knowledge of benefits, (2) limited access to activity programmes and facilities, (3) lack of support, (4) low self esteem, and (5) concerns over personal safety [26,28].

Older adults who engage in a wider spectrum of activities (comprising physical, cognitive and socialising components) are less likely to develop dementia than those that engage in only one type of activity or no activity at all [2]. We therefore developed a complex activity intervention known as the ThinkingFit programme that included design elements to overcome the known barriers to activities and included specific physical, cognitive and social activities associated with reduced dementia risk. To prepare participants for new activities and facilitate substantial lifestyle changes, we included an early pre-phase Do-Something-Different Everyday (DSD) behavioural programme that has been used successfully to alter lifestyle behaviours in diverse populations [29]. Here were report our findings from the pilot and feasibility study of this intervention for a future randomized controlled trial in MCI.

## Methods

### Participants

Patients from two local memory clinics were screened for possible inclusion if they had received a diagnosis of MCI. MCI was diagnosed in these clinics by a consensus panel (two experienced old age psychiatrists and a neuropsychologist) based on a full psychiatric assessment, physical examination with an emphasis on neurological examination and a neuropsychological test battery. The clinics use either the Addenbrooke’s Cognitive Examination Revised (ACE-R; [30]) or Revised Cambridge Cognitive Examination (CAMCOG-R; [31]). We completed the Mini-Mental State Examination for all participants recruited (MMSE; [32]).

Inclusion criteria were:

1. A consensus diagnosis of MCI [33].

2. Sedentary lifestyle with no regular participation in physical exercise defined as two or three times a week for at least 20 minutes duration, or participation in active organised sport more than once a week, in the previous six months.

3. At low risk from serious adverse effects from increased physical activity as indicated by the revised Physical Activity Readiness Questionnaire (PAR-Q; [34]).

The criteria defining a sedentary lifestyle were used to identify particularly inactive individuals to ensure that the feasibility of the intervention was examined in the MCI population that may benefit most from increased activity.

Exclusion criteria for patients with MCI:

1. Type 1 (insulin dependent) diabetes mellitus.

2. Blood pressure above 160 mmHg systolic or 100 mmHg diastolic.

3. Body weight more than 140% of ideal body weight.

4. Musculoskeletal or other medical problems preventing safe participation in regular moderate intensity exercise. This included a resting tachycardia (heart rate above 100 BPM) and history of myocardial infarction or unstable angina within the last month.

Participants with modifiable exclusion criteria were reconsidered after successful management. Participants taking medications affecting heart rate had to be on a stable dosing regime for 3 months prior to commencing the study in order to control for potential spurious results on fitness measures caused by these treatments.

### Study design

For this open label study participants served as their own controls. Data were collected at T0, T1 and T2. These three time points divided the study period into the control period (T0 to T1) and intervention period (T1 to T2).

#### Control period and early pre-phase

**Figure 1:**
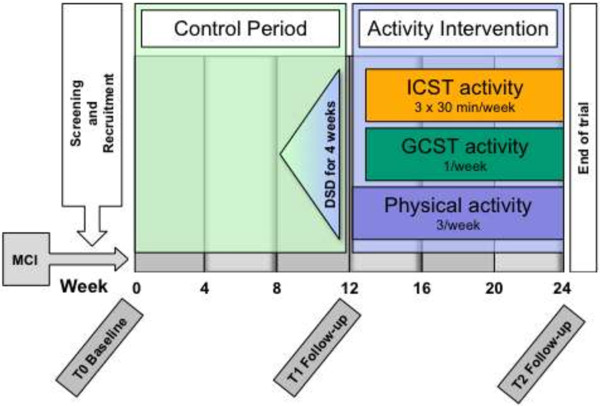
Study design.

The control period was between T0 and T1. The DSD activities were presented in an early pre-phase, during the final four weeks before T1, with the aim of increasing behavioural flexibility in anticipation of participating in 5.5 hours of activities per week (Figure 1). A choice of 36 DSD activities were presented in a booklet and participants were asked to complete an activity everyday for four weeks prior to starting the ThinkingFit activities. Following completion, participants recorded the date and location, made comments, and assigned a mark on a 4-point Likert scale (I enjoyed doing this activity: I strongly agree/agree/disagree/disagree strongly) to record compliance.

#### Intervention period

The 12-week ThinkingFit programme followed the second assessment at T1. The programme consisted of the three activities: (1) physical activity, (2) group based cognitive stimulation training (GCST) and (3) individual cognitive stimulation training (ICST). A description of the activities follows below and they are summarised in Table 1.

1) Physical activity

Participants were asked to complete a minimum of three, 30-45 min physical activity sessions per week at moderate heart rate intensity. The default physical activity was unsupervised walking from home, and participants unable to walk exercised using an upright exercise bike. Moderate heart rate intensity training zones (65-77% of maximum heart rate, estimated to be less than 60% of VO2 max) were determined for each participant from their predicted maximum heart rate (HR_max_ = 220–age). During exercise, participants received sound and vibration feedback from a heart rate monitor (Oregon Scientific, model SE102), that alerts the participant when heart rate is outside the preset parameters to help them train within a predetermined training intensity zone. Heart rate monitors were worn on the wrist and measured electrocardiographic signals via a chest strap. Participants also wore an electronic data logger (Oregon WM100). The data logger collected continuous heart rate data to provide session feedback to participants and to record the number of walks. Because all but one of the participants walked for exercise we will describe the related methods in detail. At the initial supervised home visit, the fitness instructor assessed the suitability of footwear and clothing for the activity. They provided the participant with an A2 sized poster that contained visual and written reminders on completing a five-minute warm-up before walking and on applying and operating the equipment mentioned above. The poster also contained a calendar where planned supervision sessions dates were entered and activity completion data recorded by the participant. The instructor then walked a route with the participant that had been identified using ordinance maps. They conducted a risk assessment of the route whilst walking with the participant to ensure safe participation. Participants were encouraged to exercise continuously for 30 minutes minimum as far as possible, whilst taking care to rest if they felt over exerted. They were encouraged to extend the duration of their walk to 45 minutes as their exercise tolerance increased. During supervised walks the instructor taught the participant how to judge and maintain their effort using the Borg Scale of perceived exertion [35]. Participants were offered 7 home visits for supervision, at set intervals (sessions 1, 2, 5, 8, 16, 24, 32) with more frequent visits initially to facilitate adherence. They were also offered telephone contact as required to promote adherence. Patients taking beta-blocker medication exercised at a heart rate equivalent to a specific effort (14 on the Borg scale) as these medications lower heart rate and result in suboptimal training when training intensity zones are calculated as described above [35,36].

2) Group based cognitive stimulation training (GCST)

GCST took place in groups of around 8 participants. During a weekly 2.5 hr session, adult education classes in arts and crafts were provided by experienced tutors in the following format: (1) Greeting and orientation–15 min, (2) Introduction and first part of activity–30 min, (3) Break–15 min, (4) Second part of activity–60 min, (5) Feedback and relaxation–30 min. The cognitive stimulation potential of education activities were optimised by emphasising surprise, variety and multi-sensory stimulation. Sample activities included pottery, painting, cooking, tap-dancing, playing brass instruments, rope craft, genealogy, British sign language, digital photography and drawing. Adherence to the programme was encouraged by providing appropriate supervision and structure, and by diversifying activities and settings. The nature of activities was not disclosed until they were started in order to reduce selection of only familiar or desirable activities, which could otherwise reduce engagement and negate the cognitively stimulating effects of novelty. The activities were delivered in a way that made them accessible to persons with cognitive impairment, by: (1) providing detailed explanations, (2) presenting information in more than one format, (3) wearing name tags, (4) presenting information at a slower pace, (5) emphasising hands-on participation, (6) providing individual support, and (7) keeping group sizes to around 8 participants. These were implemented by providing additional training and materials to tutors.

3) Individual cognitive stimulation training (ICST)

Participants were asked to take part in ICST 3 times per week for a minimum of 30 minutes. Training was aimed at improving specific cognitive functions such as attention, speed of processing, working memory, problem solving and reasoning. Training took place on the Lumosity programme (Lumos Labs, Inc.; San Francisco) that offers different games and puzzles and provides continuous feedback of performance and suggests games and puzzles to ensure balanced training. The feasibility of this programme has been demonstrated in older adults with MCI [37]. Participants could access this training at local community centres or at home, and received training and supervision from a tutor.

**Table 1:** Comparison of the DSD, physical, GCST and ICST activities

**Activity**	**Duration**	**Number of sessions offered**	**Number of sessions supervised**	**Session duration**	**Setting**
**Do Something Different (DSD)**	4 weeks, in early pre-phase	36	None	Variable	Home or community
**Physical**	12 weeks	36	7	30-45 mins	Home or community
**Group based cognitive stimulation (GCST)**	10 weeks	10	10	2.5 hours	Community centre
**Individual cognitive stimulation (ICST)**	10 weeks	30	10	30 mins	Community centre or home

Participants consented to the implementation of evidence based psychological motivational techniques to improve their adherence to the activities [38]. The study was approved by the Essex 1 Research Ethics Committee (09/H0301/64) and the research was completed in accordance with the Helsinki Declaration. All participants provided written informed consent.

### Outcome measures

The main feasibility outcome measures were recruitment and retention rates. Adherence to the activities was also studied because it is an important determinant of the success of the interventions. Adherence to the activities was measures as percentage confirmed completion of the offered (1) 28 DSD activities (2) 36 physical activities, (3) 10 GCST sessions, and (4) 10 supervised ICST sessions. Confirmation of activity completion was established by feedback entries (date, location, comments, 4-point Likert scale) for DSD activities, directly observation for the physical, GCST and ICST activities, and also by data from the logger and entries on the calendar on the participant poster for physical activities.

Repeated physical, neuropsychological and quality of life measures were administered at baseline (T0), after 6 to 12 weeks of treatment as usual for the control condition (T1), and after 12 weeks of participating in the intervention programme (T2).

Physical health outcomes included resting heart rate, blood pressure and body mass index (BMI). Resting heart rate and blood pressure were measured after 10 minutes sitting in a quite location, usually in the morning and participants were asked to refrain from drinking caffeine containing drinks, smoking or exercising beforehand. Participants then had their mass and height measured, and their BMI calculated (BMI = (mass in kg) / (height in m^2^)) using an online calculator [39]. Cardiovascular fitness was measured using resting heart rates and recovery heart rates via a modified Siconolfi Step Test [40]. The original test has a step height of 10 inches but we found that this often raised heart rates above 77% of predicted maximum heart rate in older adults, making it unsuitable for sub-maximal fitness testing. We therefore decreased the height to 6-inches (152 mm) and participants stepped up and down a bench for three minutes, completing 17 (step up and down) cycles per minute. Heart rate (HR) was measured during the step-test, immediately after completion and at one minute. The test was paced using a metronome to ensure standardised application. This sub-maximal stress test has a very low risk when combined with an exercise safety questionnaire such as the revised PAR-Q, and is therefore safe in community settings.

Neuropsychological outcome measures included the Halstead Trail Making test (TMT) parts A and B, verbal- and category- fluency, and digit span forwards and backwards. These measures had been validated or used in MCI populations [41-44]. Life quality was measured on the World Health Organization Quality of Life (WHOQOL)–BREF and the Alzheimer’s Disease Cooperative Study MCI Activities of Daily Living Scale (ADCS-MCI-ADL [43,45]).

### Statistical analysis

All participants who started the activity intervention were included in the primary analysis. Treatment effects were analysed using repeated measures ANOVA with Huynh-Feldt correction where sphericity was violated. Pairwise comparisons were conducted using a Bonferroni correction for multiple comparisons. Analyses were conducted in SPSS 18 for PC.

## Results

**Figure 2:**
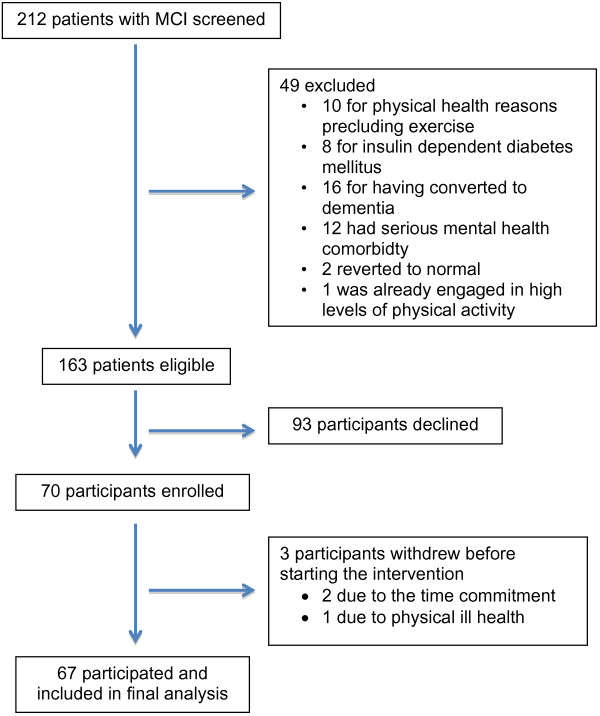
Flowchart of screening and recruitment.

212 patients with MCI were screened from two memory clinics. 163 cases were eligible; exclusions (n = 49) were as follows: 10 cases were excluded for physical health reasons precluding exercise, 8 for insulin dependent diabetes mellitus, 16 had converted to dementia, 12 had serious mental health comorbidity, 2 reverted to normal and 1 was already engaged in high levels of physical activity (Figure 2).

Seventy participants enrolled (mean age 73.7 years; 41 male), 67 started the programme and were included in the analysis, and 63 completed more than 50% of the activities offered. Three participants dropped out due to the required time commitment and ill health. Activity adherence rates were high: DSD activities = 83%; physical activity = 71%; GCST = 83%; ICST = 67%. Participants received a mean of 4.70 telephone contacts (SD = 2.89, range 1-13). One participant used a stationary exercise bike for the physical activity whilst the rest walked. The mean duration of exercise recorded by the data loggers was longer than the recommended minimum of 30 minutes (Mean = 36.3 mins, SD = 8.6 mins). The intervention was provided to 10 groups across 5 different locations. Table 2 shows descriptive statistics for completers and decliners.

**Table 2:** Mean age in years and MMSE scores for persons who completed the intervention and those who declined to take part

	**Completers**	**Decliners**	***p***
***N***	67	93	
**Age (SD)** *	73.9 (8.3)	76.4 (6.8)	.10
MMSE (SD)*	26.3 (2.6)	26.4 (2.3)	.73
**Gender (% male)** *	58	49	.32

*Pre-screening MMSE and age data were available for only 52 of those who declined and 61 participants who agreed to take part.

Significant treatment effects for which T2 showed improvements over both T0 and T1 were evident on physical health measures (decreased body mass index and systolic blood pressure), fitness measures (decreased resting and recovery heart rate), and cognition (backwards digit span; see Table 3). Other treatment effects, which showed an improvement at T2 compared to one but not both of T0 and T1, were evident for quality of life, letter fluency and forward digit span.

**Table 3:** Comparisons between control and intervention conditions on physical, cardiovascular, cognitive, functional and life quality measures

**Physical health measures**						
	***N***	**T0**	**T1**	**T2**	**F**	***p***
Systolic BP (mmHg)	53	145.1 (14.2)^a^	144.4 (15.0)^b^	135.8 (15.7)^a,b^	17.1	<.001
Diastolic BP (mmHg)	53	75.4 (16.2)	78.2 (9.4)	74.3 (9.0)	3.1	.07
BMI (kg/m^2^)	52	26.3 (3.6)^a^	26.2 (3.5)^b^	25.9 (3.4)^a,b^	7.0	<.005
**Cardiovascular fitness measures**						
Resting heart rate (beats/min)	53	68.2 (10.7)^a^	69.6 (13.1)^b^	64.6 (10.2)^a,b^	11.0	<.001
1 minute recovery heart rate (beats/min)	52	74.8 (14.0)^a^	75.6 (15.2)^b^	68.8 (11.8)^a,b^	14.2	<.001
**Cognitive measures**						
Forward digit span	43	9.4 (2.1)^a^	9.5 (2.4)	10.0 (2.3)^a^	3.4	<.05
Backwards digit span	43	5.3 (2.1)^a^	5.3 (2.0)^b^	6.3 (2.3)^a,b^	8.6	<.001
TMT-A (sec)	41	50.0 (21.1)	45.5 (19.3)	45.9 (19.3)	2.6	.09
TMT-B (sec)	43	184.5 (138.0)	192.0 (137.0)	146.3 (124.9)	2.6	.09
Letter fluency	43	14.1 (4.8)^a^	14.6 (7.3)	16.3 (5.7)^a^	3.4	.053
Category fluency	43	12.7 (4.6)^a^	10.5 (4.5)^a,b^	13.0 (4.7)^b^	7.8	.001
**Quality of life and functional abilities**						
WHO-QOL total	52	256 (38)	252 (43)^a^	263 (37)^a^	3.3	<.05
ADCS-MCI-ADL	53	45.2 (4.9)	44.7 (5.4)	45.5 (7.6)	0.5	.54

The table shows data for the control period (T0 to T1) and intervention period (T1 to T2). Data are mean (standard deviation). a and b denote pairs that differ significantly at *p* < .05.

Two serious adverse events were recorded (one stroke, one fracture of ankle) but appeared unrelated to the intervention and did not occur during activity participation.

## Discussion

The results from this study demonstrate the feasibility of recruiting and safely engaging elderly patients with MCI in a complex activity intervention. We achieved high recruitment and activity adherence rates from elderly patients with low pre-existing levels of physical activity. Adherence rates compared favorably with those from studies in MCI that had required less activity time per week or had shorter intervention periods, with rates of 63-79% reported [8-10,46]. The high recruitment rates may be the result of the lack of effective interventions to reduce the risk of dementia in this patient group, resulting in increased interest in intervention studies. Furthermore, the high levels of supervision and support provided may have been attractive to this group who are inactive, and contributed to the high adherence rates despite the 5.5 hours per week required to complete the activities.

In this age group, the majority of patients are not sufficiently physically active [5]. In our cohort, only one person was excluded due to existing adequate physical activity and the majority of patients with MCI may therefore benefit from activity programmes. Studies of other behavior change methods to increase physical and cognitive activity in over 50s have been proposed and these include combined goal setting and mentoring via telephone [13].

The intervention resulted in the expected improvements in physical fitness whilst cognitive outcome measures revealed either stable performance during the control period with improvement following the intervention period (backward- digit span, letter fluency), or deterioration during the control period with stability or improvement following the intervention (TMT-A, TMT-B, category fluency; Table 3). The control period results are expected in the natural progression of neurodegenerative disorders where performance in some cognitive domains remaining stable whilst others decline. Therefore, in MCI, effective interventions may change these trajectories and respectively either result in improvements or stability. The improvement demonstrated on cognitive measures is in line with the findings from meta analysis of the effects of increased physical fitness on cognition in older adults, which is evident despite the relatively short duration of the intervention [15], and improved cognition has also been reported in more recent studies in MCI following physical activity [8,9] and more tentatively for cognitive activity interventions [46]. Together with improvement reported on quality of life measures, our findings indicate that a relatively brief period of activity participation already results in improvement on cognitive outcomes in MCI.

The design of the activity programme addressed the known factors that limit adherence and maintenance described by other researchers [47]. Engagement was promoted by using specific psychological techniques to facilitate behaviour change, and we took advantage of group dynamics to improve motivation, enjoyment and engagement. Adherence was promoted by using skilled instructors who tailored physical activities to individuals’ needs and practical circumstances. The high adherence rates are therefore likely the result of addressing previously highlighted design limitations. Known limitations of self-reporting of physical activity completion were addressed by employing continuous time and heart rate data capture during physical activities thereby improving the reliability of the findings.

The risk of falls is increased in older adults and can be exaggerated by brisk walking [27,48]. We did not encounter any falls and very few serious adverse events, most likely because properly performed and facilitated exercise can reduce the risk of falls [49]. In comparison, a significant increase in falls was reported in a large community based activity prescription study that encouraged walking but did not provide home based activity facilitation and face-to-face support for walking [27]. These results suggest that face-to-face support and activity facilitation at home may reduce the risk of falls related to increased walking for older adults with MCI.

The reasons for the three patients dropping out were the required time commitment and ill health, which are in keeping with other studies [47]. The intervention required substantial time and effort on behalf of patients and carers. Despite this, patients reported improved quality of life, which suggests that participation was considered worth the effort.

The MCI sample was a heterogeneous group that likely included the most prevalent causes of dementia such as Alzheimer’s disease, cerebrovascular disease and Lewy body disease. Whilst this heterogeneity may be a limitation for other preventative interventions that target specific disease processes this is not the case for physical activity interventions because lower incidences for both the most prevalent causes of dementia and all other causes are associated with regular physical activity [7,50-52].

The major limitation of this intervention is the relatively high cost; however, it is low in comparison to dementia care costs and any dementia risk reduction will likely reduce the cost of care. A potential limitation in generalizing our results is that the intervention took part in only one county. However, Essex is fairly representative of England as a whole as it comprises rural, suburban and town areas, where all social classes are represented.

We employed a multimodal activity intervention because combinations of physical and cognitive activities appear more beneficial than either alone in older adults [53], and naturalistic studies have shown that combinations of social, cognitive and physical activities reduced the risk of dementia compared to only one of these elements [2]. Our methods did not allow analyses to determine the relative contribution of the different activities to the results and a future randomized controlled trial using different intervention arms will be required for this. In addition, we were not able to determine the contribution from the DSD activities on the results because it was introduced in the pre-phase. The improvements on physical health, fitness and cognitive measures are unlikely the direct results of the DSD activities considering their varied stimulation and brief duration. DSD activities however likely contributed significantly to the high adherence rates due to their behavior change potential.

Other complex multimodal activity based dementia prevention studies are currently underway. A systematic review identified multi-domain, randomized controlled trials by searching the Current Controlled Trials metaRegister with the terms: “Mild Cognitive Impairment” OR “prevention of dementia” OR “prevention of Alzheimer disease”. We identified two ongoing studies, the Multidomain Alzheimer Preventive Trial (MAPT; NCT00672685) and the Finnish Geriatric Intervention Study to Prevent Cognitive Impairment and Disability (FINGER; NCT01041989). These differ from our methods as they target older adults who are either healthy, or at risk of dementia but cognitively normal.

## Conclusions

MCI patients who are at high risk of dementia and relatively elderly can be recruited and safely engaged in a complex multimodal activity intervention developed to reduce the risk of dementia, and it therefore appears feasible to conduct a randomized controlled trial to examine the effects of long term participation on rates of conversion to dementia from MCI.

## Competing interests

No competing interests, financial or otherwise arise from this research.

## Authors’ contributions

TD was the lead investigator and grant holder. He designed the study and co-wrote this manuscript. MC was the principal investigator, assisted with designing the study, recruited and assessed all the participants, supervised other research staff and helped prepared this manuscript. TJW analysed the data and co-wrote the manuscript. BCF assisted with designing the study and in particular the DSD aspect, and helped prepare the manuscript. TS assisted with assessing and recruiting participants and commented on the drafts of the manuscript. ZW assisted with the design of the study and recruitment and co-wrote the manuscript. All authors read and approved the final manuscript.

## Pre-publication history

The pre-publication history for this paper can be accessed here:

http://www.biomedcentral.com/1471-244X/14/129/prepub
